# Genomic pedigree reconstruction identifies predictors of mating and reproductive success in an invasive vertebrate

**DOI:** 10.1002/ece3.5694

**Published:** 2019-10-02

**Authors:** Brenna A. Levine, Marlis R. Douglas, Amy A. Yackel Adams, Björn Lardner, Robert N. Reed, Julie A. Savidge, Michael E. Douglas

**Affiliations:** ^1^ University of Arkansas Fayetteville Arkansas; ^2^ U.S. Geological Survey Fort Collins Colorado; ^3^ Colorado State University Fort Collins Colorado; ^4^ Present address: University of Tulsa Tulsa Oklahoma

**Keywords:** *Boiga irregularis*, Brown Treesnake, ddRAD, parentage, reproductive ecology, single nucleotide polymorphisms

## Abstract

The persistence of an invasive species is influenced by its reproductive ecology, and a successful control program must operate on this premise. However, the reproductive ecology of invasive species may be enigmatic due to factors that also limit their management, such as cryptic coloration and behavior. We explored the mating and reproductive ecology of the invasive Brown Treesnake (BTS*: Boiga irregularis*) by reconstructing a multigenerational genomic pedigree based on 654 single nucleotide polymorphisms for a geographically closed population established in 2004 on Guam (*N* = 426). The pedigree allowed annual estimates of individual mating and reproductive success to be inferred for snakes in the study population over a 14‐year period. We then employed generalized linear mixed models to gauge how well phenotypic and genomic data could predict sex‐specific annual mating and reproductive success. Average snout–vent length (SVL), average body condition index (BCI), and trappability were significantly related to annual mating success for males, with average SVL also related to annual mating success for females. Male and female annual reproductive success was positively affected by SVL, BCI, and trappability. Surprisingly, the degree to which individuals were inbred had no effect on annual mating or reproductive success. When juxtaposed with current control methods, these results indicate that baited traps, a common interdiction tool, may target fecund BTS in some regards but not others. Our study emphasizes the importance of reproductive ecology as a focus for improving BTS control and promotes genomic pedigree reconstruction for such an endeavor in this invasive species and others.

## Graphical abstract

Our study details the use of genomic tools (i.e., double‐digest restriction site‐associated DNA sequencing) to develop novel genomic resources (>6,000 SNPs) for a high‐profile and invasive vertebrate pest (i.e., Brown Treesnake). We used a subset of these SNPs to reconstruct a multigenerational genomic pedigree for a wild population of Brown Treesnakes from which we inferred patterns and predictors of annual mating and reproductive success, and we juxtaposed phenotypes of individuals with high annual mating and reproductive success against those of commonly trapped individuals to assess existing management efficacy. Our results promote the use of genomic pedigree reconstruction to elucidate the reproductive ecology of invasive species with cryptic coloration and behaviors, so as to inform and improve management.

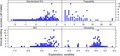

## 1 INTRODUCTION

Species declines and extinctions are driven by multiple factors, the most egregious being anthropogenic habitat alteration (Travis, 2003), species introductions (Vitousek, Mooney, Lubchenco, & Melillo, 1997), and climate change (Thomas et al., 2004). Introduced species, in particular, have provoked negative responses in a variety of ecological contexts: community assembly (Sanders, Gotelli, Heller, & Gordon, 2003), competitive exclusion/niche displacement (Mooney & Cleland, 2001), interspecific hybridization/introgression (Muhlfeld et al., 2009), and even natural selection (Strauss, Lau, & Carroll, 2006). Introductions are deemed second only to human‐induced habitat loss as a major cause of species endangerment (Simberloff, 2001), yet are the primary cause of global avian extinctions (Clavero & García‐Berthou, 2005). Within a more social context, invasive species also impact global economies (Olson, 2006) and human health (Juliano & Lounibos, 2005).

Reproductive ecology is a key element in the establishment and persistence of an invasive species. Those that exhibit high fecundity not only increase their probability of establishment but also mitigate the potential for an Allee effect and/or issues that stem from demographic and environmental stochasticity (Lockwood, Hoopes, & Marchetti, 2013). Following invasion, a species also must be able to persist and cope with changes in an alien environment. These responses are mediated through the mating system (e.g., selfing, monogamy, promiscuity), its characteristics (e.g., traits that promote mating and reproductive success), and associated reproductive phenomena (e.g., inbreeding, multiple paternity) that influence genetic variation and evolutionary potential (Ellegren & Galtier, 2016).

Two reproductive ecology parameters critical to development of successful invasive species control are the number of offspring that an individual produces annually (referred to here as annual reproductive success; ARS) and the number of mates with which an individual produces offspring annually (referred to here as annual mating success; AMS). Quantification of average ARS in an invasive species yields an important estimate of the annual ability of a population to replenish itself. Further, when phenotypes associated with high ARS can be identified, these can be juxtaposed with phenotypes of individuals removed by existing control methods to gauge the efficacy of management and identify areas for improvement. Differently, the number of mates with which an individual produces offspring can have implications for maintenance of genetic diversity (Ellegren & Galtier, 2016), as production of offspring with multiple mating partners increases the overall genetic variation present in the resulting offspring (Foerster, Delhey, Johnsen, Lifjeld, & Kempenaers, 2003). In this regard, juxtaposition of phenotypes associated with high AMS against those of individuals targeted by control can also provide information regarding the potential effect of control methods on genetic variation and evolutionary potential over time.

Here, we applied these concepts to explore the reproductive ecology of the Brown Treesnake (*Boiga irregularis*; BTS), introduced to Guam from the island of Manus in the Admiralty Archipelago during or shortly after World War II and subsequently deemed one of the “world's worst” invasive species (Lowe, Browne, Boudjelas, & De Poorter, 2000). Despite limited propagule pressure (≤10 individuals; Richmond, Wood, Stanford, & Fisher, 2014), its population size reached two million by the 1980s (Fritts & Rodda, 1998). BTS have since caused considerable ecological changes, to include extirpation/extinction of 10 native bird species (Savidge, 1987) and population declines of endemic nonavian vertebrates (Rodda, Fritts, & Chiszar, 1997). Not surprisingly, this decline in biodiversity has had a cascading effect on community dynamics and structure (Caves, Lambers, Tewksbury, & Rogers, 2013; Mortensen, Dupont, & Olesen, 2008; Rogers, Lambers, Miller, & Tewksbury, 2012). The BTS invasion has also been detrimental to the economy (Perry & Vice, 2009) and has implications for human health (Fritts, McCoid, & Haddock, 1990).

Birth rate is a demographic parameter fundamental to population persistence (Cole, 1954), and thus, an in‐depth understanding of reproductive ecology should be a focus of control efforts for BTS and other invasive species. Not surprisingly, this theme has been amplified in the BTS literature (e.g., Engbring & Fritts, 1988; Jordan & Rodda, 1994; Moore et al., 2005; Rodda, Fritts, McCoid, & Campbell, 1999; Siegel, Aldridge, Clark, Poldemann, & Gribbins, 2009). However, the cryptic behavior of BTS constrains field studies and stymies in‐depth research on its reproduction in the wild (Greene & Mason, 1998; Kahl, Henke, Hall, & Britton, 2012; Mathies, Franklin, & Miller, 2004; Trembath & Fearn, 2008). This is unfortunate in that successful control and eradication hinges on the ability to eliminate breeding individuals more rapidly than they are replenished (Rodda et al., 2002). If phenotypes associated with elevated mating and reproductive success can be so targeted, then the potential for management to achieve this goal is enhanced considerably.

Fortunately, ARS and AMS can be inferred from genomic pedigrees, as reconstructed from DNA samples. However, genetic markers for BTS are limited, particularly with regard to fine‐grained estimates of relatedness [but see Richmond et al. (2014) and Unger et al. (2015)]. This capacity has been expanded as of late through derivation of single nucleotide polymorphisms (SNPs) that are not only cost‐effective but also highly applicable to nonmodel organisms (Ekblom & Galindo, 2011).

Our central goal was to reconstruct a multigenerational genomic pedigree for BTS that would allow patterns of mating and reproductive success to be inferred in the wild. To do so, we juxtaposed genome‐wide SNPs identified from double‐digest restriction site‐associated DNA (ddRAD) libraries against phenotypic and genomic data collected over a 14‐year period for 426 BTS from a geographically closed population on Guam. Predictors of AMS and ARS were identified and the genetic mating system of BTS characterized. The results of this study can be applied to assess the efficacy of existing BTS control and to improve management. In a more general sense, this study highlights the importance of understanding invasive species reproductive ecology in the context of management action and promotes the use of genomic pedigree reconstruction to achieve this goal.

### 1.1 Hypotheses

We postulated that both sexes are promiscuous (i.e., producing offspring with more than one mating partner per year) as this is the most common snake mating system (Rivas & Burghardt, 2005). We predicted that four factors, each focusing on a different aspect of BTS ecology, were related to AMS and ARS. Specifically, we predicted that individual AMS and ARS would be influenced by the following: (a) snout–vent length (SVL; the length from the tip of the snout to the cloacal vent), (b) body condition index (BCI; a measure of body mass relative to SVL), (c) trappability (a measure of the propensity to enter baited traps; Le Cœur et al., 2015), and (d) the degree to which an individual is inbred.

We predicted an influence of SVL on AMS and ARS because SVL is a trait correlated with AMS (Shine et al., 2000) and ARS in other snakes (Levine et al., 2015). Male snakes with larger SVLs have a competitive advantage over smaller males in combat for gaining priority‐of‐access to females (Duvall & Schuett, 1997; Madsen & Shine, 1993, 1994), and such “combat dances” have been observed among male BTS in a laboratory setting (Greene & Mason, 2000). Differently, females with larger SVLs may appear more attractive to males (Blouin‐Demers, Gibbs, & Weatherhead, 2005), resulting in greater AMS, and their larger body cavities may correlate with an increased capacity to produce eggs (Blouin‐Demers et al., 2005; Brown & Shine, 2007). We expected body condition to be influential for AMS and ARS in that underweight individuals in either sex may lack sufficient energy reserves to search for mates (Lind & Beaupré, 2015), engage in mating and mating‐related activities [e.g., male combat (Shine et al., 2000)], and/or produce offspring (Aubret, Bonnet, Shine, & Lourdais, 2002).

We also postulated that trappability [i.e., the propensity to enter baited traps (Le Cœur et al., 2015)] would impact AMS and ARS for both sexes for two reasons. First, trappability may serve as a proxy for risk‐taking behaviors (Boyer, Réale, Marmet, Pisanu, & Chapuis, 2010; Réale, Gallant, Leblanc, & Festa‐Bianchet, 2000; Wilson, Coleman, Clark, & Biederman, 1993). In this sense, positive correlations exist between boldness/exploratory behavior and trappability across taxa (Biro & Dingemanse, 2009). We predicted that individuals with high trappability would display greater values for AMS and ARS in that they would be more likely to take risks regarding mate searching and/or acquisition. Second, those individuals may also enter traps more often due to enhanced olfactory capabilities that improve their capacity to find baited traps (Shivik, 1998; Shivik & Clark, 1997). This should also promote mating and reproductive success in that olfaction influences mate finding in male BTS (Greene, Stark, & Mason, 2001; Mathies, Levine, Engeman, & Savidge, 2013). Importantly, we recognize the complexity of the relationship between trappability, AMS, and ARS, and thus simply offer a rationale for their association. Finally, we predicted that a negative relationship would be found between AMS/ARS and the degree to which the focal individual is inbred. This would represent an echo of the founder effects manifested by BTS on Guam (Richmond et al., 2014).

## 2 MATERIALS AND METHODS

### 2.1 Study site

The study site is a 5‐ha enclosure on Andersen Air Force Base (northern Guam) that was fenced in 2004 to prevent immigration/emigration of BTS (Rodda, Savidge, Tyrrell, Christy, & Ellingson, 2007; Tyrrell et al., 2009). We collected tissue samples (blood, tail clips, and ventral scale clips) from 426 unique individuals (217 females, 207 males, and two of unknown sex) over an eight‐year span (2009–2017). We back‐extrapolated median estimated hatch dates (assuming a hatch size of 350 mm SVL) for each individual from sex‐specific growth rates and SVL at first capture that ranged from years 2002 to 2016. From 2004 to 2018, we captured BTS in baited traps or by hand during nocturnal visual searches along maintained transects. Baited traps deployed in the enclosure were similar to those used in operational control on the island. Over the course of the study, data including SVL, mass, and method of capture were collected from individuals each time they were encountered.

### 2.2 ddRAD library preparation

We extracted genomic DNA using the QIAamp Fast DNA Tissue Kit (QIAGEN^©^) and quantified concentrations with a Qubit 2.0 Fluorometer (Invitrogen, Inc.), following manufacturer protocols. We verified the presence of high‐quality genomic DNA (i.e., molecular weight > 10kb) by separating a 5‐µl aliquot of each extract on a 2% agarose gel for 50 m at 100 mV, with visualization via GelGreen on a blue‐light transilluminator (Gel Doc™ EZ Imager; Bio‐Rad). We prepared extracted DNA samples using a ddRAD protocol (Peterson, Weber, Kay, Fisher, & Hoekstra, 2012) subsequently modified in Bangs, Douglas, Mussmann, and Douglas (2018; Appendix S1) for single‐end sequencing (100 bp length) on an Illumina HiSeq 4000.

### 2.3 Bioinformatics

We inspected fastq files for quality using *FastQC* (Andrews, 2014). We used the *process_radtags* module of *Stacks 2.0* (Catchen, Amores, Hohenlohe, Cresko, & Postlethwait, 2011; Catchen, Hohenlohe, Bassham, Amores, & Cresko, 2013) to demultiplex reads by individual barcode with default values for score limit (*s* = 10) and sliding window size (*w* = 0.15). We clustered raw reads into loci using *Stacks 2.0*, which uses three main parameters to cluster reads: minimum number of identical sequencing reads to be considered a putative locus (=*m*), maximum number of nucleotide differences within each locus (stack) per individual (=*M*), and maximum number of nucleotide differences between individuals at a locus (=*n*; Catchen et al., 2011). We determined the correct parameters for clustering reads into loci by following published protocols (Paris, Stevens, & Catchen, 2017; Rochette & Catchen, 2017). The correct values of these parameters were revealed by parameter optimization to be as follows: *m* = 3; *M* = 2; *n* = 2 (Appendix S1).

We then used *Stacks 2.0* to cluster raw reads from all samples, with 75 selected for catalog construction (Rochette & Catchen, 2017) to span the entire sampling period, include only high‐coverage individuals (mean number of reads/locus = 26.67 ± 9.69), and minimize potential batch effects that could stem from digestion, ligation, and sequencing procedures. We excluded those individuals sequenced more than once for quality control (Appendix S1). Upon completion of the core modules (*ustacks*, *cstacks*, *sstacks*, *tsv2bam*, *gstacks*), we used the *populations* module to retain only those loci present in at least 95% of individuals (*r* = 0.95). To minimize linkage disequilibrium, we also only retained the first SNP at each locus *(‐‐write_single_snp*).

### 2.4 Pedigree reconstruction

We used the *R* package *Sequoia* (Huisman, 2017) to iteratively reconstruct a maximum‐likelihood multigenerational pedigree from SNP genotypes, sex data, and estimated birth years. *Sequoia* is optimized for SNP data sets, jointly considers a variety of alternative relationship categories (e.g., grandparents/grand‐offspring in additional to parents/offspring), and allows the consideration of more than two generations at a time. Due to the longitudinal nature of our study and the presence of multiple overlapping generations of BTS in the study population, this last point is essential as multiple generations of snakes can feasibly produce offspring in the same cohort, thereby making it impossible to declare a priori a clear candidate parent group for each cohort [as is required by other parentage analysis software such as *Colony* (Jones & Wang, 2010)]. To prepare the SNP data set for pedigree reconstruction, we first used *plink 1.9* (Purcell et al., 2007) to test for and discard loci in linkage disequilibrium (LD) and out of Hardy–Weinberg equilibrium (HWE). For LD and HWE tests, we considered all individuals (*n* = 24) born from 2002 to 2004 (2004 = year of fence construction) as founders. We tested for LD with the *‐‐indep* function, evaluating 50 SNP windows, five SNPs at a time, and with a variance inflation factor (VIF) cutoff = 2.

The *Sequoia* user manual recommends retaining only those loci with a minor allele frequency (MAF) ≥0.3 in the population, and to tweak this parameter value and the level of LD tolerated until a set of 300–700 SNPs is achieved. Rather than compromising the stringency of LD that was tolerated in our data set and risking potential nonindependence of SNPs, we chose a MAF = 0.3 for the population which resulted in 654 SNPs for pedigree reconstruction.

We accomplished initial parentage assignments with the genotype file and a life history file with *MaxSibiter* = 0. This allowed us to scan the pedigree for obvious errors, as well as for duplicates accidentally retained. We altered the parameter data frame (=*Specs*) to increase *MaxSibshipSize* = 100, *MaxSibiter* = 40, and *UseAge*=“Extra.” The *agepriors* file was modified to prevent impossible parentage assignments (e.g., one‐year‐old parents; J. Huisman, personal communication, 9 April 2018). This was accomplished by changing the one‐year‐old prior for maternity from 0.035 to 0.000 and the one‐year‐old prior for paternity from 0.001 to 0.000. All other parameters were kept at default. We constructed the full pedigree by setting the altered parameter file as the *SeqList*. We ran *Sequoia* using *R v. 3.4.3* (R Core Team, 2013).

We assessed the accuracy of our reconstructed pedigree and the ability of our SNP data set to correctly identify familial relationships in three ways. First, we used *Sequoia's EstConf* function to calculate confidence probabilities of parentage assignments for dams and sires of known ID (Table S1). We ran simulations for 50 iterations (*nSim* = 50) and assumed that 40% of parents were not sampled (*ParMis* = 0.4), and we found all probabilities of parentage assignments to known individuals ranged from 0.93 to 0.99. Second, we regressed pairwise genomic relatedness [estimated from the entire set of SNPs (*N* = 6,180) using the *QG89_avg* estimator in the *R* package *irelr* (Gonçalves da Silva & Russello, 2011)] on to pairwise pedigree relatedness [estimated from the reconstructed *Sequoia* pedigree including dummy individuals using the *R* package *Pedantics* (Morrissey & Wilson, 2010)]. We found a strong correlation between estimated pairwise genomic relatedness and estimated pairwise pedigree relatedness (Pearson correlation coefficient = 0.71; Figure S1) that was also within the range of correlations previously reported for data sets analyzed with *Sequoia* (=0.47–0.81; Huisman, 2017). Third, we tested the ability of *Sequoia* to correctly identify duplicate individuals by running it on a data set with genotypes of 114 individuals that were sequenced twice in different lanes following separate library preparations (Appendix S1), and found that all duplicates were correctly flagged. We ran *Sequoia* and *irelr* with *R v. 3.4.3*, and we ran *Pedantics* in *RStudio* (RStudio Team, 2015) with *R v. 3.5.0*.

We parsed the *Pedigree* file generated by *Sequoia* to calculate AMS and ARS for each individual for each calendar year that they were known to be alive, and we included dummy parents assigned by *Sequoia* in estimates of AMS. Breeding is largely aseasonal in BTS on Guam (Savidge, Qualls, & Rodda, 2007), although there is some evidence that peak copulation occurs in the dry season with hatching occurring in the following wet season (least rain occurs in March; Rodda & Savidge, 2007). Using calendar years as cutoffs for AMS and ARS is thus somewhat arbitrary but consistent among individuals, a fact which should make our analyses more conservative. Fourteen individuals were assigned a known individual as one parent but neither a known nor dummy individual as their other parent. We included these assignments in estimates of AMS and ARS so as to not underestimate ARS, and preliminary analyses without these assignments demonstrated that their inclusion had no effect on the results of downstream analyses.

### 2.5 Statistical analyses

We fit generalized linear mixed models (GLMMs) for each sex separately to test predictors of AMS and ARS using the *glmer* function of the *R* package *lme4* (Bates, Maechler, Bolker, & Walker, 2015). We excluded from analyses records for two individuals of unknown sex, as well as annual records for which AMS and ARS > 0 but also for which phenotypic data were incomplete or lacking (=11 annual female records and 10 annual male records). These exclusions removed data collected during year 2014 from analyses. The final data set comprised records of AMS and ARS for years 2004–2013 and 2015–2018.

For both males and females, we analyzed a complete data set that included all annual data records for individuals (hereafter “complete”; data collected during years 2004–2013 and 2015–2018) and a data set filtered by mean annual SVL to only include data collected when individuals were likely adults (hereafter “SVL‐filtered”; data collected during years 2005–2013 and 2015–2018). BTS can mature over a large size range (Savidge et al., 2007), so drawing a clear cutoff by age or SVL at which snakes are deemed sexually mature is, at best, difficult and, at worst, risks biasing the data set by excluding from analyses legitimate adults that matured earlier than is average or including juveniles that matured later. Nevertheless, we recognize that our inclusion of juvenile records in the complete data sets may be problematic, particularly if traits undergo ontogenetic shifts [e.g., change in trappability as BTS mature (Rodda et al., 2007)]. Therefore, we also analyzed sex‐specific data sets that we filtered to include only data collected when individuals were likely adults (=“SVL‐filtered”). Savidge et al. (2007) reported that most females and males reach sexual maturity at SVLs between 910 and 1,025 mm and between 940 and 1,030 mm, respectively. Thus, these SVL‐filtered data sets only included annual records that exceeded these cutoffs (i.e., mean female SVL ≥ 910 mm, mean male SVL ≥ 940 mm). Filtering the data set by mean annual SVL also ensured that only fully trappable snakes were included in analyses of these data (Tyrrell et al., 2009).

**Figure 1:**
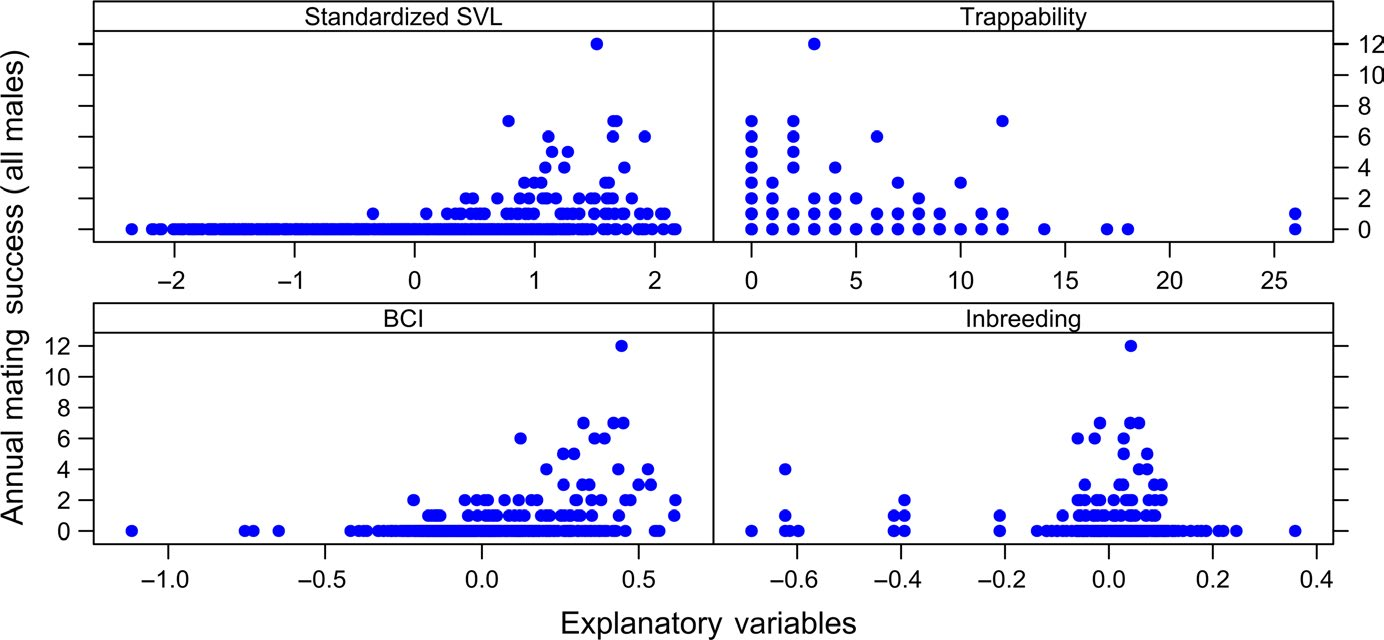
Distribution of four annual measurements with respect to annual mating success (i.e., number of mates with which an individual produced offspring over the course of a calendar year) for male Brown Treesnakes (*Boiga irregularis*) collected over a 14‐year period from a geographically closed 5‐hectare population on Guam. Annual measurements include standardized average snout–vent length (“Standardized SVL”), number of times the individual was captured in a baited trap (“Trappability”), average body condition index (=BCI), and degree of genome‐wide inbreeding (“Inbreeding”). There were a total of 661 annual observations of 207 males

**Figure 2:**
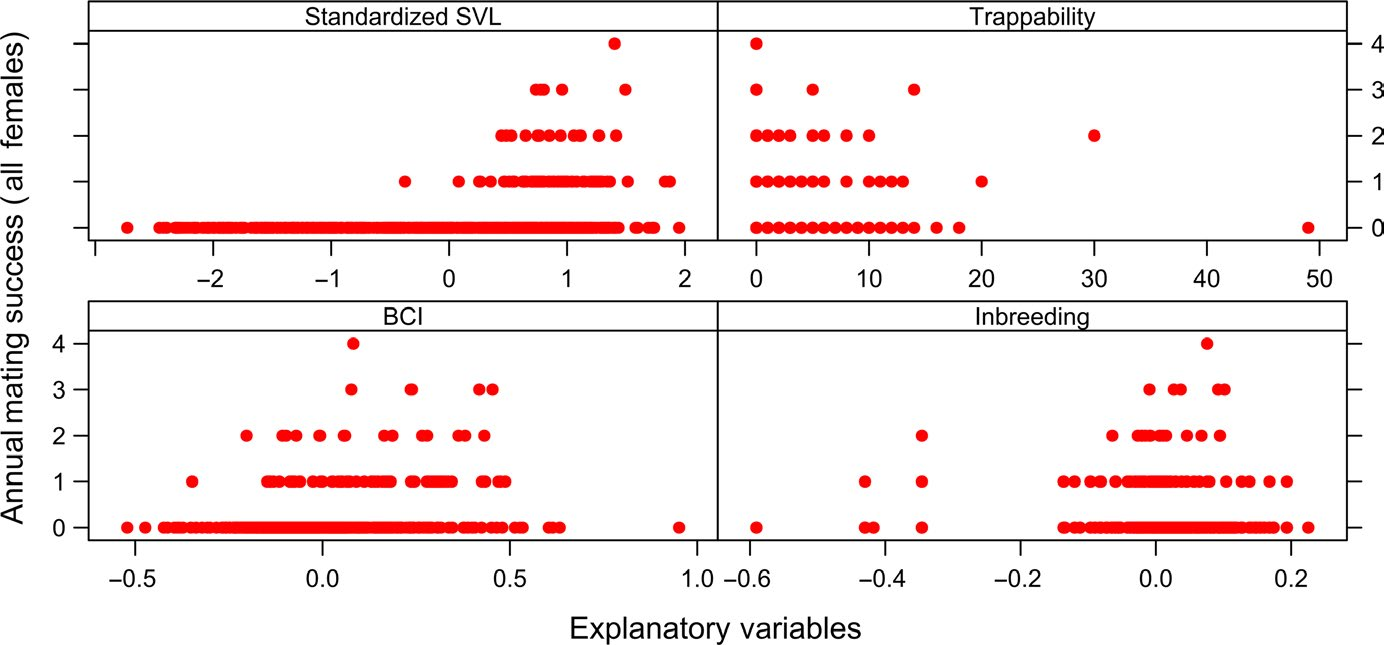
Distribution of four annual measurements with respect to annual mating success (i.e., number of mates with which an individual produced offspring over the course of a calendar year) for female Brown Treesnakes (*Boiga irregularis*) collected over a 14‐year period from a geographically closed 5‐hectare population on Guam. Annual measurements include standardized average snout–vent length (“Standardized SVL”), number of times the individual was captured in a baited trap (“Trappability”), average body condition index (“BCI”), and degree of genome‐wide inbreeding (“Inbreeding”). There were a total of 735 annual observations of 217 females

**Figure 3:**
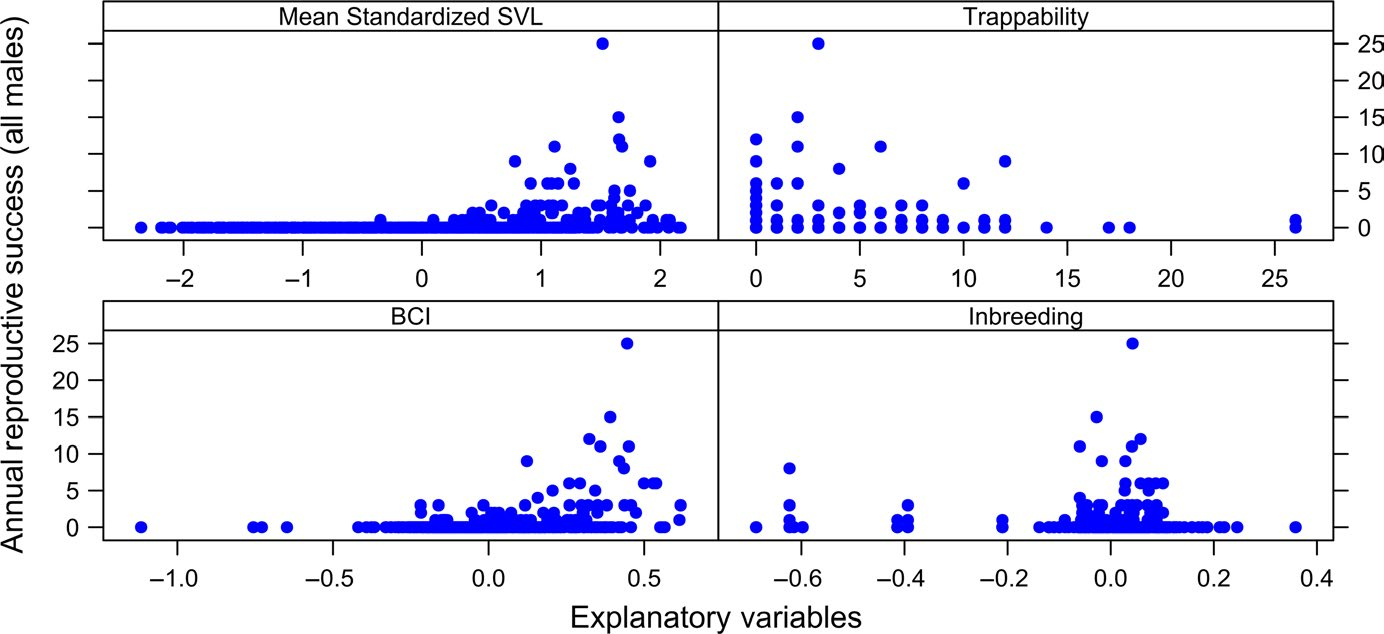
Distribution of four annual measurements with respect to annual reproductive success (i.e., number of offspring an individual produced over the course of a calendar year) for male Brown Treesnakes (*Boiga irregularis*) collected over a 14‐year period from geographically closed 5‐hectare population on Guam. Annual measurements include standardized average snout–vent length (“Standardized SVL”), number of times the individual was captured in a baited trap (“Trappability”), average body condition index (“BCI”), and degree of genome‐wide inbreeding (“Inbreeding”). There were a total of 661 annual observations of 207 males

**Figure 4:**
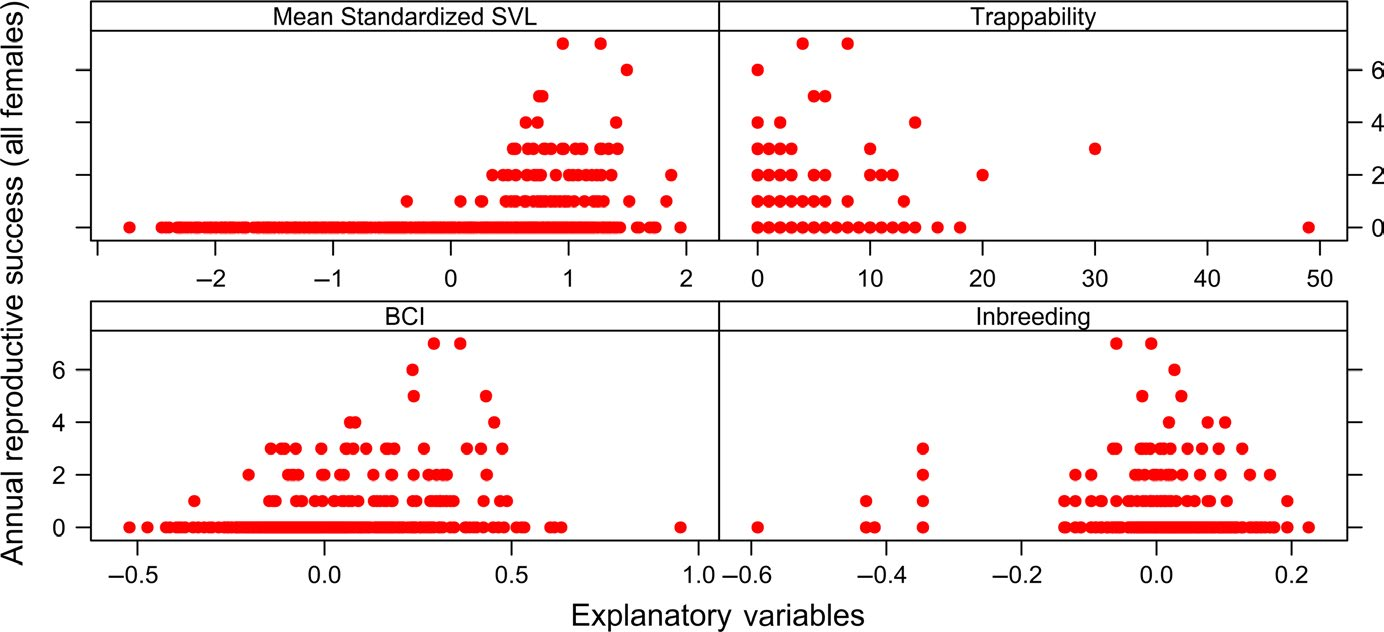
Distribution of four annual measurements with respect to annual reproductive success (i.e., number of offspring an individual produced over the course of a calendar year) for female Brown Treesnakes (*Boiga irregularis*) collected over a 14‐year period from a geographically closed 5‐hectare population on Guam. Annual measurements include standardized average snout–vent length (“Standardized SVL”), number of times the individual was captured in a baited trap (“Trappability”), average body condition index (“BCI”), and degree of genome‐wide inbreeding (“Inbreeding”). There were a total of 735 annual observations of 217 females

We used plots to visualize the structure and distribution of all data sets prior to fitting the models (complete data sets shown in Figures 1, 2, 3, 4). We assayed for the potential presence of interactions among explanatory variables by generating coplots and tested for collinearity among explanatory variables (Zuur, Ieno, & Elphick, 2010). To do so, we visually inspected correlation matrices and calculated VIFs with the *R* package *MCtest* (Imdadullah, Aslam, & Altaf, 2016), with no evidence of significant collinearity among explanatory variables [all VIFs < 2 (Zuur et al., 2010)].

We did not model interactions among explanatory variables in sex‐specific GLMMs of AMS and ARS for several reasons. First, we had no a priori expectation that explanatory variables would interact to influence AMS or ARS (Harrison et al., 2018). Second, visualization of potential interactions with coplots did not indicate strong interactions among predictor variables (Bolker et al., 2009; Zuur & Ieno, 2016; Zuur et al., 2010). Third, we avoided overparameterization of our models by not including interaction terms (Harrison et al., 2018).

We specified a Poisson error distribution with a log‐link for GLMM analyses of the complete and SVL‐filtered sex‐specific data sets because of its utility for count data (Zuur, Ieno, Walker, Savaliev, & Smith, 2009) and the means of our response variables (i.e., *µ* < 5; DuVal, 2012). We chose not to convert our data to zeros (=AMS/ARS = 0) and ones (=AMS/ARS > 0) for modeling with a binomial distribution instead as this resulted in extreme parameter estimates and nonrandom patterns within our residuals. We validated our choice of distribution and method of modeling our data for final reduced models by testing for overdispersion (all *p* > .05), generating Q‐Q plots, and plotting scaled residuals against predicted values [simulated with the *DHARMa* package in *R* (Hartig, 2017)], with all models compliant. A Poisson distribution has also been used previously to model predictors of numbers of offspring produced in other organisms [e.g., male eastern chipmunks (Patterson & Schulte‐Hostedde, 2011)].

We modeled sex‐specific AMS and ARS as linear functions of four fixed and two random effects. Fixed effects included annual mean BCI, annual mean SVL, annual trappability, and a genome‐wide estimate of inbreeding [*F*_hat3_ (Yang, Lee, Goddard, & Visscher, 2011)]. Random effects included individual and year (Bolker et al., 2009). We included the “individual” random effect to account for repeated measurements of individuals and the “year” random effect to accommodate temporal variation over the course of the study (e.g., variable numbers of traps deployed per night each year).

We estimated annual mean BCIs by taking the residuals of the regression of natural log‐transformed annual mean body mass versus natural log‐transformed annual mean SVL separately for each sex (Schulte‐Hostedde, Zinner, Mllar, & Hickling, 2005). We assessed annual trappability by summing the number of times an individual was caught in a baited trap in a given year [see Réale et al. (2000) and Le Cœur et al. (2015)]. Average annual SVL was calculated for each individual, and this variable was standardized to have a mean of zero and a standard deviation of one to facilitate convergence of GLMMs (Harrison et al., 2018). Finally, we used *PLINK 1.9* to derive the genome‐wide estimate of inbreeding (*F*_hat3_, Yang et al., 2011) for each individual from the final filtered set of SNPs (*N* = 654). After running the global models, we used the *drop1{stats}* function in *R* (R Core Team, 2013) to test the significance of fixed effects using likelihood ratio tests of the global model against a null model lacking the predictor of interest (*χ* ^2^, *α* = .05; see Nystrand, Cassidy, and Dowling (2018) and Sales et al. (2018) for similar approaches). To avoid possible bias of effect sizes, we only report estimates and standard errors for parameters for the global models (Harrison et al., 2018; Tables 1 and 2). Statistical analyses were conducted in *RStudio* (*R v. 3.5.0*; RStudio Team, 2015).

**Table 1:** Results for sex‐specific generalized linear mixed models (GLMMs) of annual mating success (AMS) for Brown Treesnakes (*Boiga irregularis*) from a geographically closed population on Guam. GLMMs were run for (A) complete sex‐specific data sets (male = 661 records; female = 735 records) and (B) sex‐specific data sets filtered by SVL to include only likely adult records (male = 312 records; female = 367 records). Sex‐specific AMS was modeled as a linear function of four annual fixed effects (“Parameter”): average body condition index (“BCI”), standardized average snout–vent length (“SVL”), the number of times the individual was captured in a baited trap (“Trappability”), and the individual's genome‐wide estimate of inbreeding (“Inbreeding”). GLMMs also included individual and year of sampling as random effects (not shown). GLMMs employed a Poisson error distribution with a log‐link

Fixed effect	Male AMS				Female AMS			
	Estimate	*SE*	LRT	*p*	Estimate	*SE*	LRT	*p*
(A) Complete								
BCI	3.717	0.910	15.496	**<.001**	1.184	0.738	2.613	.106
SVL	1.643	0.275	65.820	**<.001**	1.483	0.245	63.988	**<.001**
Trappability	0.095	0.033	8.635	**.003**	0.034	0.020	2.640	.104
Inbreeding	0.368	1.168	0.099	.753	−0.607	1.073	0.310	.578
(B) SVL‐filtered								
BCI	3.909	0.974	15.108	**<.001**	0.868	0.736	1.408	.235
SVL	1.416	0.394	13.105	**<.001**	0.794	0.351	5.000	**.025**
Trappability	0.091	0.034	7.441	**.006**	0.028	0.020	1.921	.166
Inbreeding	0.510	1.188	0.185	.667	−0.525	1.038	0.248	.618

Note

Significance of fixed effects was assessed with likelihood ratio tests of the global model containing the effect of interest against a null model without the effect, with significant *p*‐values in bold (*χ* ^2^, *α* = .05). Estimates and standard errors are reported for effects in the global model to avoid overestimation of effect sizes.

Abbreviations: Estimate, statistical value; LRT, log‐likelihood ratio; *p*, *p*‐value; *SE*, standard error.

**Table 2:** Results for sex‐specific generalized linear mixed models (GLMMs) of annual reproductive success (ARS) for Brown Treesnake (*Boiga irregularis*) from a geographically closed population on Guam. GLMMs were run for (A) complete sex‐specific data sets (male = 661 records; female = 735 records) and (B) sex‐specific data sets filtered by SVL to include only likely adult records (male = 312 records; female = 367 records). Sex‐specific ARS was modeled as a linear function of four annual fixed effects (“Parameter”): average body condition index (“BCI”), standardized average snout–vent length (“SVL”), the number of times the individual was captured in a baited trap (“Trappability”), and the individual's genome‐wide estimate of inbreeding (“Inbreeding”). GLMMs also included individual and year of sampling as random effects (not shown). GLMMs employed a Poisson error distribution with a log‐link

Fixed effect	Male ARS				Female ARS			
	Estimate	*SE*	LRT	*p*	Estimate	*SE*	LRT	*p*
(A) Complete								
BCI	4.194	0.938	19.240	**<0.001**	1.723	0.736	5.616	**0.018**
SVL	1.755	0.290	68.804	**<0.001**	1.683	0.262	78.852	**<0.001**
Trappability	0.113	0.034	11.730	**<0.001**	0.049	0.020	5.623	**0.018**
Inbreeding	0.404	1.388	0.085	0.771	−0.582	1.219	0.223	0.636
(B) SVL‐filtered								
BCI	4.365	1.004	18.393	**<0.001**	1.369	0.734	3.548	0.060
SVL	1.601	0.415	14.971	**<0.001**	1.013	0.365	7.556	**0.006**
Trappability	0.113	0.036	10.810	**0.001**	0.044	0.020	4.664	**0.031**
Inbreeding	0.621	1.439	0.187	0.666	−0.505	1.138	0.194	0.660

Note

Significance of fixed effects was assessed with likelihood ratio tests of the global model containing the effect of interest against a null model without the effect, with significant *p*‐values in bold (*χ* ^2^, *α* = 0.05). Estimates and standard errors are reported for effects in the global model to avoid overestimation of effect sizes.

Abbreviations: Estimate, statistical value; LRT, log‐likelihood ratio; *p*, *p*‐value; *SE*, standard error.

## 3 RESULTS

### 3.1 ddRAD sequencing and bioinformatic processing

Illumina sequencing of ddRAD libraries resulted in 1,590,917,152 raw reads, with mean number/individual = 3,734,547 (standard deviation [*SD*] = ±1,114,685.18). When considering samples duplicated for quality control, sequencing resulted in 1,971,406,088 raw reads (*µ* = 3,650,752 reads ± 1,105,292.25). Mean sequencing coverage/individual = 25.97*x* (±8.26), while mean coverage/individual = 24.80*x* (±8.32), including duplicates.

After clustering raw reads into loci and filtering with the *populations* module of *Stacks 2.0*, we identified 6,180 SNPs, each present in at least 95% of sequenced BTS (*N* = 426). Of the 6,180 SNPs that passed initial filtering, we discarded 217 due to departures from HWE (*p* < .05), 482 with evidence of LD (VIF > 2), and 4,827 that had allele frequencies < MAF threshold (=<0.3). We used all remaining loci (*n* = 654) for pedigree reconstruction.

### 3.2 Pedigree reconstruction

We were able to assign 69 known females as dams to 199 known individuals and 51 known males as sires to 257 known individuals with confidence probabilities ranging from 0.93 to 0.99 (Table S1). A promiscuous mating system was evident in that both sexes produced offspring via multiple mates each year. Of individuals that reproduced, males produced offspring with an average of 2.28 mates/year (±2.06), whereas females produced offspring with an average of 1.33 mates/year (±0.64). Of these individuals, mean male ARS = 3.38 (±4.03) offspring/year, while mean female ARS = 2.01 (±1.33).

### 3.3 GLMMs of AMS and ARS

We analyzed 1,396 complete annual data records, to include 661 records for males and 735 for females collected during years 2004–2013 and 2015–2018. The SVL‐filtered data sets included 312 records for likely adult males and 367 records for likely adult females collected during years 2005–2013 and 2015–2018. Mean age of reproduction was 3.68 (±1.09) and 3.94 (±1.20) years for males and females, respectively. Mean *F*_hat3_ was 0.02 (±0.11) and ranged from −0.69 to 0.36.

We found BCI, SVL, and trappability to have significant positive effects on male AMS when considering both the complete data set and the SVL‐filtered data set for males (Table 1A,B). When considering the complete and SVL‐filtered data sets for females, only SVL had a significant positive effect on AMS (Table 1A,B). BCI, SVL, and trappability had significant positive effects on ARS when analyzing both the complete and SVL‐filtered data sets for males (Table 2A,B). SVL, BCI, and trappability also had positive significant effects on female ARS when considering the complete female data set (Table 2A), with only significant positive effects of SVL and trappability on female ARS remaining when analyzing the SVL‐filtered data set (Table 2B).

## 4 DISCUSSION

### 4.1 Characterization of the BTS mating system

Our expectation of a promiscuous mating system for BTS on Guam was supported; both sexes produced offspring with multiple mates each year. Promiscuity is the most common type of mating system in snakes (Rivas & Burghardt, 2005), although polygyny (multiple mating only by males) and polyandry (multiple mating only by females) are also prevalent (Duvall, Schuett, & Arnold, 1993; Kissner, Weatherhead, & Gibbs, 2005).

For individuals that produced offspring in a given year, we found mean ARS for males and females to be 3.38 (±4.03) and 2.01 (±1.33), respectively, and these data provide important insight into annual BTS reproductive capacity. Mean clutch size has been a difficult parameter to estimate on Guam, with few wild clutches located, and low annual recruitment (as estimated from appearance of hatchlings) has been reported for the study population [i.e., 0.5 female offspring per female per year (Rodda & Savidge, 2007)]. Gravid females have previously been found to contain 3–12 eggs when palpated (Rodda & Savidge, 2007), and Savidge et al. (2007) estimated mean clutch size to be 4.3 eggs (±2.2). Yet, this value was based off of counts of ovarian follicles and oviductal eggs rather than eggs laid by females. A few gravid females have been collected in the wild on Guam and taken into the laboratory for parturition, resulting in clutches ranging from 3 to 11 eggs (Savidge et al., 2007). However, successful hatching of eggs can be variable. For example, of three Guam females induced to reproduce in a laboratory setting, the number of eggs hatched was 0 (out of 3 eggs laid), 2 (out of 8 eggs laid), and 7 (out of 10 eggs; Mathies & Miller, 2003). In this regard, our estimate of female ARS in particular may be a more informative metric for BTS reproductive ecology than clutch size, as it represents the realized annual reproductive success of females and not a count that may include unfertilized or unhatched eggs. Importantly, these estimates represent minimum mean AMS and ARS for males and females as it is possible that some hatchlings died before they could be sampled.

### 4.2 Predictors of AMS and ARS

We predicted that AMS and ARS in both sexes would be influenced by SVL, BCI, trappability, and degree of genome‐wide inbreeding. We found significant effects of SVL, BCI, and trappability on AMS for males, and a significant effect of SVL on AMS for females regardless of the data set analyzed (Table 1A,B). Further, we found significant effects of SVL, BCI, and trappability on both male and female ARS when analyzing the complete data sets, with significance remaining for these effects when the SVL‐filtered data sets were analyzed, save the effect of BCI on female ARS (Table 2A,B).

The effect of SVL on AMS and ARS for males and females was present regardless of the data set analyzed (i.e., complete vs. SVL‐filtered). Larger SVLs have previously been shown in male snakes to correlate with greater AMS (Shine et al., 2000) and ARS (Levine et al., 2015) due to larger males having a competitive advantage over smaller males for gaining priority‐of‐access to females (Duvall & Schuett, 1997; Madsen & Shine, 1993, 1994). While male–male combat for priority‐of‐access to females has not been observed in wild BTS, it has been observed in captivity (Greene & Mason, 2000). Larger females, on the other hand, may reflect adequate energy reserves for production of offspring (Aubret et al., 2002) and, given this, appear more attractive to males (Blouin‐Demers et al., 2005), thereby facilitating offspring production with more partners. Furthermore, larger females have larger body cavities which may correlate with an increased capacity to produce offspring (Blouin‐Demers et al., 2005; Brown & Shine, 2007). An additional factor that may contribute to our results is that males and females with larger SVLs are older, and therefore are more likely to have reached sexual maturity and to participate and in mating and reproduction. This is supported by a stronger effect of SVL on AMS and ARS for both males and females when analyzing the complete (as opposed to the SVL‐filtered) data sets (Tables 1 and 2).

We predicted that BCI would influence AMS and ARS, and found this to be true for males regardless of the data set analyzed. Males with better body condition have greater energy reserves, and this should support a variety of mating‐related behaviors. For example, larger male BTS gain access to females by being successful in confrontations with smaller males (Greene & Mason, 2000). Sufficient energy reserves also permit mate searching by males (Lind & Beaupré, 2015) and allow more time for mate searching in that competing activities such as foraging are less mandatory (Beaupré, 2008). Although the energetic status of male snakes often influences their ability to mate [e.g., Red‐Sided Garter Snake (Shine & Mason, 2005), Timber Rattlesnake (Lind & Beaupré, 2015)], the relationship between BCI and male reproductive success remains somewhat nebulous (Shine & Mason, 2005). Male snakes seemingly contribute little energy to actual reproductive success, in that gamete production requires limited energy (Aubret et al., 2002). However, they do expend considerable energy during related activities (Lind & Beaupré, 2015; Shine & Mason, 2005). It is possible that the significant effect of BCI on ARS we identified is due to factors associated with mate acquisition, or even the physical ability of males to mate [e.g., adequate plasma testosterone levels (Bonnet & Naulleau, 1996); elevated corticosterone levels due to food stress (Waye & Mason, 2008)]. Indeed, chronic stress and elevated corticosterone levels are associated with low BCI in BTS (Waye & Mason, 2008), whereas elevated male BCIs are related to higher levels of plasma testosterone (Mathies, Cruz, Lance, & Savidge, 2010), and both elevated corticosterone and reduced plasma testosterone negatively affect reproduction in male BTS (Aldridge, Siegel, Bufalino, Wisniewski, & Jellen, 2010; Moore et al., 2005). Male BCI may also have the capacity to directly influence reproductive success. Male body size is associated with testes mass, such that larger male BTS may in fact have greater rates of sperm production, and thus an increased capacity for fertilization (Mathies et al., 2010).

We did not find a significant effect of BCI on female AMS regardless of the data set analyzed. Further, there was a significant effect of BCI on female ARS only when analyzing the complete data set and not the SVL‐filtered data set. We found the inconsistent effect of BCI on female AMS and ARS surprising as adequate energy reserves should be necessary for females to participate in mating and produce offspring. However, females may actively forage while vitellogenic, so as to acquire energy for reproduction [i.e., income breeding (Bonnet, Bradshaw, & Shine, 1998)]. In support of this hypothesis, Savidge et al. (2007) found that 79% of female BTS captured on Guam with vitellogenic follicles had prey in their stomachs. Therefore, a lack of a consistent effect of BCI on female AMS and ARS could be due to the presence of income breeding in BTS. Future work will be required to determine why BCI is important to male AMS and ARS, but seemingly less so to female AMS and ARS.

We also predicted that trappability would influence AMS and ARS in both sexes. With regard to AMS, we only found a significant positive effect for males (although these estimates were small for both the complete and SVL‐filtered data sets; Table 1A,B). These results indicate that males with a greater propensity to enter baited traps mate with more partners to produce offspring. Importantly, although juveniles are not considered trappable by baited trap (Rodda et al., 2007), the significant effect of trappability on male AMS remained when the male records were filtered by SVL to retain only those for likely adults. Trappability has often been used as a proxy for propensity of individuals to engage in risk‐taking behaviors (Biro & Dingemanse, 2009). Presumably, males bold enough to enter baited traps will also take risks to acquire mates. For instance, movement by bold individuals in search for mates may increase their risk of predation, while those less bold would not, with the likelihood of encountering potential mates subsequently diminished (Sih, Bell, & Johnson, 2004).

Trappability is also related to the ability of an individual to detect chemical stimuli from baited traps. BTS use olfactory cues to find prey in baited traps (Shivik, 1998), and male BTS also use olfactory cues (i.e., female pheromones) to find mates (Greene et al., 2001; Parker, Patel, Zachry, & Kimball, 2018) and elicit courtship (Greene & Mason, 1998). Therefore, it is likely that males with better chemosensory abilities will not only find baited traps more frequently but also have greater success in finding and acquiring mates. This may be the reason that we found a relationship between trappability and AMS in males but not females. The use of olfactory cues to detect prey in baited traps may also contribute to both males and females with greater trappability having greater ARS. Individuals that are better able to detect prey in baited traps may also be better at energy acquisition which would result in greater energy availability for mating and reproduction. However, additional research will be required to untangle the complicated relationship between trappability and ARS.

We found the degree to which an individual was inbred to have no effect on AMS or ARS for males or females, and these results were consistent regardless of the data set analyzed. This finding was surprising because most individuals (68.4%) in our study had estimates of genome‐wide inbreeding (*F*_hat3_) > 0, with several (8.3% females; 6.8% males) consistent with having half‐sibling parental relationships (*F*_hat3_ > 0.125). In other systems, mating success in the wild is negatively impacted by the degree to which focal individuals are inbred (Janicke, Vellnow, Lamy, Chapuis, & David, 2014; Joron & Brakefield, 2003).

Although we found no significant effect on AMS or ARS for either sex, inbreeding effects may instead manifest indirectly by influencing other traits that, in turn, impact AMS and ARS [e.g., by affecting propensity for risk‐taking behavior (Richardson & Smiseth, 2017) or motivation to mate (De Boer, Eens, & Müller, 2018)]. The effects of inbreeding may also be context‐dependent, with negative impacts more pronounced under stressful conditions (Armbruster & Reed, 2005). Guam's relatively constant environment (Rodda et al., 1999) may counter environmental stress and thus act to dampen negative effects of inbreeding on AMS and ARS. Further, individuals may avoid inbreeding effects through behavioral plasticity (Lucia‐Simmons & Keane, 2015). Finally, inbreeding may simply exert minimal effects on AMS and ARS (Gooley, Hogg, Belov, & Grueber, 2017). Future work will be required to determine why inbreeding seemingly does not significantly affect AMS and ARS in Guam BTS, particularly considering the small size of the founding population (Richmond et al., 2014).

### 4.3 Implications for control

A variety of control efforts have been implemented to reduce or eliminate BTS on Guam and to prevent its dispersal to other areas (Clark, Clark, & Siers, 2018; Engbring & Fritts, 1988; Rodda & Savidge, 2007). Baited traps are a primary method of control, and improving trap success will likewise promote BTS management. Here, we interpret our results with regard to common phenotypes found in trapped individuals.

First, several studies have explored the relationship between body size and trappability (Boyarski, Savidge, & Rodda, 2008; Lardner et al., 2013; Rodda et al., 2007; Tyrrell et al., 2009), with larger individuals trapped more often than those smaller. Given this finding, our results indicate that baited traps are effective at capturing males and females that produce more offspring and males that produce offspring with more mating partners.

However, while several studies have also evaluated the relationship between trapping success and BCI, results have been variable. For example, Tyrrell et al. (2009) found a minimal positive effect of BCI on female trappability and a negative effect of BCI on male trappability, such that males with low BCIs were caught in baited traps more often. Differently, Lardner et al. (2013) found a positive correlation between BCI and accession of bait tubes for both sexes. Our study demonstrated that BCI has a positive effect on AMS and ARS for males, with a similar effect of BCI on ARS supported for females. Given these relationships between BCI and AMS/ARS, our incomplete understanding of how BCI is related to trappability (by either baited traps or bait tubes) is troubling.

Finally, it is promising that overall trappability is related to AMS for males and ARS for both males and females, although the estimates for the effects of trappability on AMS and ARS were small. Males and females with higher trappability produce more offspring annually and males with higher trappability produce offspring with more mates; importantly, these effects remain when removing likely juveniles (untrappable) from analyses. Removal of trapped individuals thus has the potential to depress the birth rate of the population by eliminating more fecund males and females. However, significant unexplained heterogeneity in trappability exists among individuals (Clark, Savarie, Shivik, Breck, & Dorr, 2012; Mason, Savidge, Rodda, & Yackel Adams, 2011; Rodda et al., 2002; Tyrrell et al., 2009), prompting concerns that trappability may have a heritable genetic component (Tyrrell et al., 2009), as documented in fishes (Cooke, Suski, Ostrand, Wahl, & Philipp, 2007). If BTS trappability is indeed heritable, then selection may yield a population with overall lower ARS, but also one that is trap‐shy (Tyrrell et al., 2009). We are currently evaluating the heritability of being trappable to gauge the potential for inadvertent adaptive responses of reproductive ecology to management action.

## 5 CONCLUSION

An understanding of the reproductive ecology of invasive species is critical for the development of effective control. Phenotypes associated with enhanced annual mating and reproductive success can be targeted to maximally impact the birth rate of the population. Furthermore, by identifying correlates of mating and reproductive success and juxtaposing them with “controllable” phenotypes, the long‐term efficacy of control can be gauged, particularly considering eco‐evo dynamics generated by the control methods themselves (Závorka et al., 2018). Here, we demonstrated the use of multigenerational genomic pedigree reconstruction as an avenue for identification of predictors of AMS and ARS in an invasive vertebrate and compared phenotypes associated with elevated AMS and ARS with those targeted by control. We did so using genomic markers that are widely applicable to nonmodel organisms (Peterson et al., 2012). These results will serve to promote similar endeavors for other invasive species.

## CONFLICT OF INTEREST

The authors declare no competing interests.

## AUTHOR CONTRIBUTIONS

All authors contributed to the conceptualization and design of the study. AAYA, BAL, BL, RNR, and JAS collected field data. AAYA and BL organized and maintained the U.S. Geological Survey Brown Treesnake database. BAL conducted all laboratory work and statistical analyses, with guidance from MRD and MED. Most editing was accomplished by MRD and MED, but all authors contributed editorial feedback.

## Data Availability

Raw sequencing reads are archived with the DRYAD data repository at https://doi.org/10.5061/dryad.47ss1b4. Data analyzed in the study are available through ScienceBase at https://doi.org/10.5066/P9X1AKVJ.
