# Supplementary material for: Genomic pedigree reconstruction identifies predictors of mating and reproductive success in an invasive vertebrate
Source: Ecol Evol. 2019 Oct 2;9(20):11863–77. doi: 10.1002/ece3.5694 (PMC6822066; doi:10.1002/ece3.5694)
Supplement: Supplementary file 1 [file ECE3-9-11863-s001.docx]

**Supporting Information for Online Publication**

**Title**: Genomic pedigree reconstruction identifies predictors of mating and reproductive success in an invasive vertebrate

**Authors**: Brenna A. Levine^1†^, Marlis R. Douglas^1^, Amy A. Yackel Adams^2^, Björn Lardner^3^, Robert N. Reed^2^, Julie A. Savidge^3^, Michael E. Douglas^1^

**Affiliations**: ^1^University of Arkansas, Fayetteville, AR 72701; ^2^U.S. Geological Survey, Fort Collins, CO 80526; ^3^Colorado State University, Fort Collins, CO 80523; ^†^University of Tulsa, Tulsa, OK 74104 (current affiliation of Brenna Levine)

**Corresponding Author**:

Dr. Brenna A. Levine

University of Tulsa

Tulsa, OK 74104

**1 | ddRAD LIBRARY PREPARATION**

Briefly, we digested 1,000–2,000 ng of genomic DNA per individual in a 30 ul reaction with 1 ul each of restriction enzymes *Msp1* and *Pst1*, 5 ul 10x CutSmart^®^ Buffer (New England BioLabs^®^ Incorporated), and 23 ul HPLC grade water. We incubated samples during digestion for 24 h at 37 °C in a Veriti 96-Well Thermal Cycler (Applied Biosystems^TM^). We confirmed successful digestion by separating 5 ul of each digest on a fresh 2% agarose gel, with visualization using GelGreen on a blue-light transluminator.

We cleaned digests with Agencourt AMPure XP beads (Beckman Coulter, Inc.) and ligated digested samples with a P1 adaptor with a unique five base pair (bp) barcode. A total of 48 unique barcodes allowed for subsequent pooling and sequencing of DNA in sets of 48 individuals (=one ddRAD library). We standardized digested DNA concentrations among samples within libraries to promote equal coverage. We performed ligation in a 30 ul reaction/individual, consisting of 22 ul digested sample, 2 ul P1 adaptor, 3 ul 10x T4 DNA ligase reaction buffer (New England BioLabs^®^ Inc.), 2 ul P2 adaptor, and 1 ul T4 DNA ligase (New England BioLabs^®^ Inc.). Ligation occurred in a Veriti 96-Well Thermal Cycler using a temperature profile of 22 °C/60 minutes, 65 °C/10 minutes, a decline of 1% ramp rate at ~1 °C per minute, and a 20 °C hold. We then pooled ligated samples in sets of 48, and performed an additional AMPure XP cleanup.

We used a Pippin Prep (Sage Science) to perform size-selection of ligated, pooled samples. We identified an appropriate fragment size range for parentage and kinship analyses by performing an *in silico* digest with *FRAGMATIC* (Chafin, Martin, Mussmann, Douglas, & Douglas, 2017). We then compared simulated numbers of fragments with results from previous *in vitro* preparation of test libraries to identify an optimal size selection (=262-350 bp).

Finally, we performed Phusion^®^ PCR on each size-selected library. This was accomplished using four 20 ul reactions per library [=5 ul size-selected DNA, 5.8 ul HPLC grade water, 4.0 ul Phusion® HF buffer (New England BioLabs® Inc.), 0.5 ul MgCl_2_ (50 mM), 0.5 ul dNTP (10 mM), 2.0 ul PCR 1 primer (2 uM), 2 ul PCR 2 indexed primer (2 uM), and 0.2 ul Phusion^®^ high-fidelity DNA polymerase (New England BioLabs^®^ Inc.]. The temperature profile for Phusion^®^ PCR in a Veriti Thermal Cycler was: 1 cycle of 98 °C/1 minute; 12 cycles of 98 °C/15 s, 62 °C/30 s, 72 °C/30 s; 1 cycle of 98 °C/7 m; and a 20 °C hold. Importantly, we ligated sets of libraries sequenced in the same lane with different indices, allowing for 96 individuals to be sequenced/lane (=2 indices x 48 barcodes). We followed Phusion^®^ PCR by a final AMPure XP cleanup, and quantified DNA concentrations using a Qubit 2.0 Fluorometer. To minimize batch and lane effects, we semi-randomly grouped DNA samples into different digestions, ligations, and sequencing runs. Additionally, to confirm batch effects did not influence our sequencing results, we sequenced 114 individuals twice in separate lanes following separate ddRAD library preparations.

**2 | STACKS PARAMETER OPTIMIZATION**

We used a *Stacks 2.0* wrapper script (*denovo_map.pl*) to test parameter settings on clustering of reads into putative loci (i.e., *M*=*n* for values equal to 1–9 while maintaining *m*=3). Once *denovo_map.pl* was completed for all parameter combinations, we re-ran the *populations* module to retain only loci genotyped in at least 80% of samples (Paris, Stevens, & Catchen, 2017). We used a script (Nicolas Rochette; bitbucket.org/rochette/rad-seq-genotyping-demo/src/) to extract loci and SNPs from each parameter value combination. We plotted three response variables against parameter value combinations for each sample subset, including: (1) loci retained by at least 80% of samples, (2) new loci added to the *Stacks 2.0* catalog and present in at least 80% of samples, and (3) distribution of SNPs per locus. We then evaluated the plots to identify the parameter values at which the number of polymorphic loci and number of SNPs shared by at least 80% of samples stabilized (Paris et al., 2017).

We performed this protocol on two different sample sets to confirm that sample selection did not impact optimization. We selected samples to be representative of the entire data set by including samples: (a) Of each tissue type, (b) collected throughout the length of the study, and (c) digested, ligated, and sequenced in different batches. We took care to ensure that no duplicates were included (see above). When performed on both sets, the protocol resulted in consistent identification of optimal clustering parameters [*m*=3, *M*=2, *n*=2].

**SUPPORTING REFERENCES**

Chafin, T. K., Martin, B. T., Mussmann, S. M., Douglas, M. R., & Douglas, M. E. (2017). FRAGMATIC: in silico locus prediction and its utility in optimizing ddRADseq projects. *Conservation Genetics Resources*, *10*(3), 325–328. doi:10.1007/s12686-017-0814-1

Paris, J. R., Stevens, J. R., & Catchen, J. M. (2017). Lost in parameter space: A road map for stacks. *Methods in Ecology and Evolution*, *8*(10), 1360–1373. doi:10.1111/2041-210X.12775

**SUPPORTING FIGURES AND TABLES**


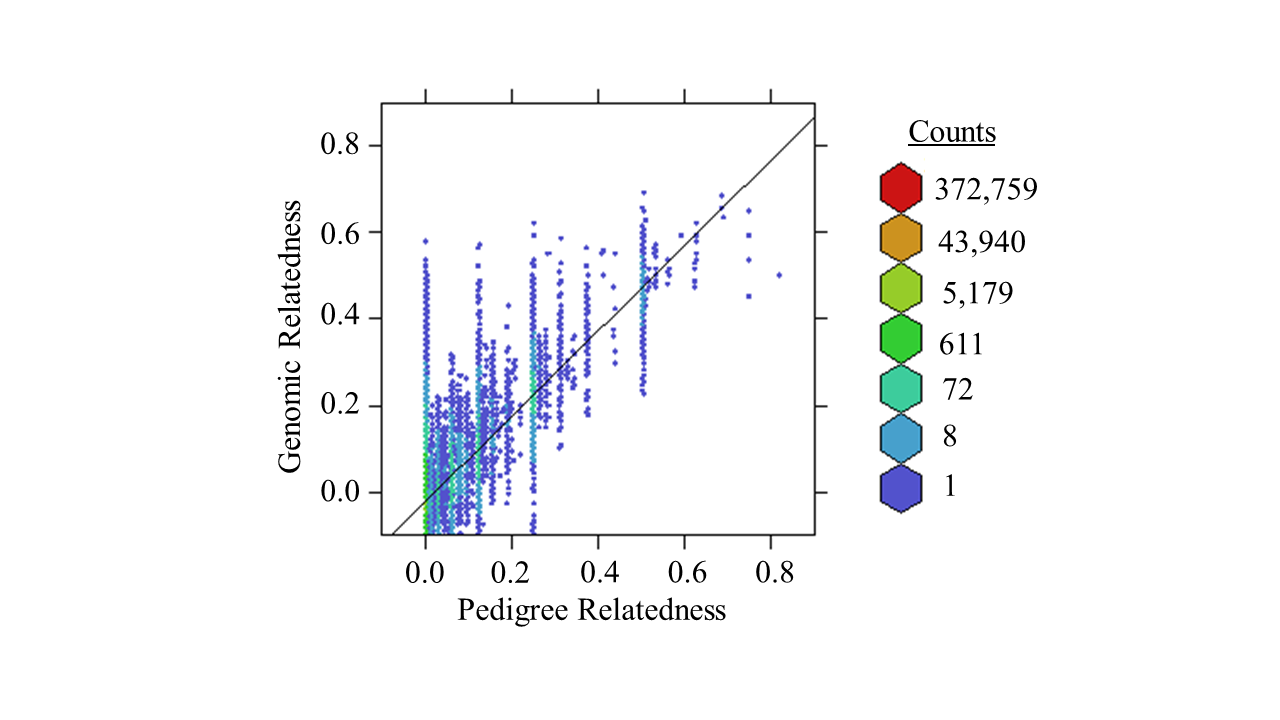


**Figure S1** To assess the accuracy the Brown Treesnake pedigree, we regressed pairwise genomic relatedness [estimated from the entire set of single nucleotide polymorphisms (SNPs; N = 6,180) using the *QG89_avg* estimator in the *R* package *irelr*] on to pairwise pedigree relatedness (estimated from the reconstructed *Sequoia* pedigree including dummy individuals using the *R* package *Pedantics*). The pedigree was constructed with 654 high minor allele frequency SNPs that were in Hardy-Weinberg Equilibrium and low linkage disequilibrium There was a strong correlation between estimated pairwise genomic and pedigree relatedness (Pearson correlation coefficient = 0.71; *p* < 0.001).

**Table S1** Confidence probabilities of parentage by dams and sires of known ID (no dummy individuals) for Brown Treesnake (*Boiga irregularis*) sampled from a geographically-closed population on Guam (N=426), as estimated with the *EstConf* function of the *R* package *Sequoia*. *EstConf* was run for 50 iterations (*nSim*=50), and simulations assumed that 40% of parents were not sampled (*ParMis*=0.4). ID=individual sample; Dam=mother of known ID; Dam Probability=probability of maternity; Sire=father of known ID; Sire Probability=probability of paternity.

| ID | Dam | Dam Probability | Sire | Sire Probability |
| --- | --- | --- | --- | --- |
| 13BTS001 | - | - | - | - |
| 13BTS002 | - | - | - | - |
| 13BTS003 | - | - | - | - |
| 13BTS004 | - | - | - | - |
| 13BTS005 | - | - | - | - |
| 13BTS006 | - | - | - | - |
| 13BTS007 | - | - | 13BTS095 | 0.99 |
| 13BTS008 | - | - | - | - |
| 13BTS009 | - | - | - | - |
| 13BTS010 | - | - | - | - |
| 13BTS011 | 13BTS039 | 0.93 | 13BTS068 | 0.99 |
| 13BTS012 | - | - | - | - |
| 13BTS013 | - | - | - | - |
| 13BTS014 | - | - | - | - |
| 13BTS015 | - | - | - | - |
| 13BTS016 | - | - | - | - |
| 13BTS017 | - | - | - | - |
| 13BTS018 | - | - | 13BTS022 | 0.99 |
| 13BTS019 | - | - | - | - |
| 13BTS020 | - | - | - | - |
| 13BTS021 | 13BTS020 | 0.97 | - | - |
| 13BTS022 | - | - | - | - |
| 13BTS023 | - | - | - | - |
| 13BTS024 | - | - | - | - |
| 13BTS025 | - | - | 13BTS103 | 0.99 |
| 13BTS026 | - | - | - | - |
| 13BTS027 | - | - | - | - |
| 13BTS028 | - | - | - | - |
| 13BTS029 | - | - | - | - |
| 13BTS030 | 13BTS125 | 0.93 | 13BTS023 | 0.99 |
| 13BTS031 | - | - | - | - |
| 13BTS032 | - | - | - | - |
| 13BTS033 | - | - | - | - |
| 13BTS034 | - | - | - | - |
| 13BTS035 | - | - | - | - |
| 13BTS036 | - | - | - | - |
| 13BTS037 | 13BTS082 | - | - | - |
| 13BTS038 | - | - | - | - |
| 13BTS039 | - | - | - | - |
| 13BTS040 | - | - | - | - |
| 13BTS041 | - | - | - | - |
| 13BTS042 | - | - | - | - |
| 13BTS043 | - | - | - | - |
| 13BTS044 | - | - | - | - |
| 13BTS046 | 13BTS082 | - | 13BTS058 | - |
| 13BTS047 | - | - | - | - |
| 13BTS048 | - | - | - | - |
| 13BTS049 | - | - | - | - |
| 13BTS050 | - | - | - | - |
| 13BTS051 | 13BTS082 | - | - | - |
| 13BTS052 | - | - | - | - |
| 13BTS053 | - | - | - | - |
| 13BTS054 | - | - | - | - |
| 13BTS055 | - | - | - | - |
| 13BTS056 | - | - | - | - |
| 13BTS057 | - | - | - | - |
| 13BTS058 | - | - | - | - |
| 13BTS059 | - | - | - | - |
| 13BTS060 | - | - | - | - |
| 13BTS061 | - | - | - | - |
| 13BTS062 | - | - | - | - |
| 13BTS063 | 13BTS082 | - | - | - |
| 13BTS064 | - | - | - | - |
| 13BTS065 | - | - | - | - |
| 13BTS066 | - | - | - | - |
| 13BTS067 | - | - | - | - |
| 13BTS068 | - | - | - | - |
| 13BTS069 | - | - | - | - |
| 13BTS070 | 13BTS020 | 0.97 | - | - |
| 13BTS072 | - | - | - | - |
| 13BTS073 | - | - | - | - |
| 13BTS074 | - | - | - | - |
| 13BTS075 | - | - | - | - |
| 13BTS077 | - | - | - | - |
| 13BTS078 | - | - | - | - |
| 13BTS079 | - | - | - | - |
| 13BTS080 | - | - | - | - |
| 13BTS081 | - | - | - | - |
| 13BTS082 | - | - | - | - |
| 13BTS083 | - | - | - | - |
| 13BTS084 | - | - | - | - |
| 13BTS085 | - | - | 13BTS087 | 0.99 |
| 13BTS086 | - | - | - | - |
| 13BTS087 | - | - | - | - |
| 13BTS088 | - | - | 13BTS087 | 0.99 |
| 13BTS089 | - | - | - | - |
| 13BTS091 | - | - | - | - |
| 13BTS092 | - | - | - | - |
| 13BTS093 | - | - | - | - |
| 13BTS094 | - | - | - | - |
| 13BTS095 | - | - | - | - |
| 13BTS096 | - | - | - | - |
| 13BTS097 | - | - | - | - |
| 13BTS098 | - | - | - | - |
| 13BTS099 | - | - | - | - |
| 13BTS100 | - | - | - | - |
| 13BTS101 | - | - | - | - |
| 13BTS102 | - | - | - | - |
| 13BTS103 | - | - | - | - |
| 13BTS104 | 13BTS125 | 0.93 | - | - |
| 13BTS105 | - | - | - | - |
| 13BTS106 | - | - | - | - |
| 13BTS107 | - | - | - | - |
| 13BTS108 | - | - | - | - |
| 13BTS109 | - | - | - | - |
| 13BTS110 | - | - | - | - |
| 13BTS111 | - | - | - | - |
| 13BTS112 | - | - | - | - |
| 13BTS113 | - | - | - | - |
| 13BTS114 | - | - | - | - |
| 13BTS115 | - | - | - | - |
| 13BTS117 | - | - | - | - |
| 13BTS118 | - | - | - | - |
| 13BTS119 | - | - | - | - |
| 13BTS120 | - | - | - | - |
| 13BTS121 | - | - | - | - |
| 13BTS122 | - | - | - | - |
| 13BTS123 | - | - | - | - |
| 13BTS124 | - | - | - | - |
| 13BTS125 | - | - | - | - |
| 13BTS126 | - | - | - | - |
| 13BTS127 | 13BTS117 | 0.97 | 13BTS019 | - |
| 13BTS128 | - | - | - | - |
| 13BTS129 | - | - | - | - |
| 13BTS130 | - | - | - | - |
| 13BTS131 | - | - | - | - |
| 13BTS132 | - | - | - | - |
| 13BTS133 | - | - | - | - |
| 13BTS134 | - | - | 13BTS002 | 0.99 |
| 13BTS135 | - | - | - | - |
| 13BTS136 | - | - | - | - |
| 13BTS137 | - | - | - | - |
| 13BTS138 | 13BTS064 | 0.93 | 13BTS072 | 0.99 |
| 13BTS140 | 13BTS075 | 0.97 | 13BTS073 | 0.99 |
| 13BTS141 | 13BTS120 | 0.93 | 13BTS022 | 0.99 |
| 13BTS142 | - | - | - | - |
| 13BTS143 | 13BTS013 | 0.97 | 13BTS102 | 0.99 |
| 13BTS144 | - | - | - | - |
| 13BTS145 | 13BTS075 | 0.97 | - | - |
| 13BTS146 | - | - | 13BTS041 | 0.99 |
| 13BTS147 | - | - | 13BTS069 | 0.97 |
| 13BTS148 | 13BTS125 | 0.93 | - | - |
| 13BTS149 | 13BTS082 | 0.93 | 13BTS031 | 0.97 |
| 13BTS150 | 13BTS115 | 0.93 | 13BTS031 | 0.97 |
| 13BTS151 | - | - | 13BTS002 | 0.99 |
| 13BTS152 | - | - | - | - |
| 13BTS153 | - | - | - | - |
| 13BTS154 | - | - | - | - |
| 13BTS155 | - | - | 13BTS073 | 0.99 |
| 13BTS156 | 13BTS060 | 0.93 | 13BTS087 | 0.99 |
| 13BTS157 | 13BTS065 | - | 13BTS041 | - |
| 13BTS158 | - | - | 13BTS086 | 0.99 |
| 13BTS159 | - | - | 13BTS002 | - |
| 13BTS160 | - | - | - | - |
| 13BTS161 | - | - | - | - |
| 13BTS162 | - | - | - | - |
| 13BTS163 | - | - | - | - |
| 13BTS164 | 13BTS016 | 0.93 | 13BTS022 | 0.99 |
| 13BTS165 | - | - | - | - |
| 13BTS166 | - | - | - | - |
| 13BTS167 | - | - | 13BTS073 | 0.99 |
| 13BTS168 | 13BTS060 | 0.93 | 13BTS084 | - |
| 13BTS169 | - | - | - | - |
| 13BTS170 | - | - | 13BTS086 | 0.99 |
| 13BTS171 | - | - | 13BTS069 | 0.97 |
| 13BTS172 | - | - | - | - |
| 13BTS173 | - | - | 13BTS102 | 0.99 |
| 13BTS174 | - | - | 13BTS069 | 0.97 |
| 13BTS175 | - | - | 13BTS101 | 0.97 |
| 13BTS176 | 13BTS039 | 0.93 | 13BTS031 | 0.97 |
| 13BTS177 | 13BTS033 | 0.93 | 13BTS069 | 0.97 |
| 13BTS178 | 13BTS013 | 0.97 | 13BTS102 | 0.99 |
| 13BTS179 | 13BTS065 | 0.93 | 13BTS069 | 0.97 |
| 13BTS180 | - | - | 13BTS073 | 0.99 |
| 13BTS181 | 13BTS020 | 0.97 | 13BTS037 | 0.99 |
| 13BTS182 | - | - | - | - |
| 13BTS183 | 13BTS082 | 0.93 | 13BTS031 | 0.97 |
| 13BTS184 | - | - | 13BTS002 | 0.99 |
| 13BTS185 | 13BTS013 | 0.97 | 13BTS091 | 0.99 |
| 13BTS186 | 13BTS039 | 0.93 | 13BTS068 | 0.99 |
| 13BTS187 | 13BTS133 | 0.97 | 13BTS101 | 0.97 |
| 13BTS188 | 13BTS120 | 0.93 | 13BTS022 | 0.99 |
| 13BTS189 | - | - | 13BTS069 | - |
| 13BTS190 | 13BTS120 | 0.93 | 13BTS022 | 0.99 |
| 13BTS191 | 13BTS080 | 0.97 | 13BTS086 | 0.99 |
| 13BTS192 | 13BTS079 | 0.97 | 13BTS073 | 0.99 |
| 13BTS193 | - | - | 13BTS022 | - |
| 13BTS194 | 13BTS197 | 0.97 | 13BTS073 | 0.99 |
| 13BTS195 | 13BTS013 | 0.97 | 13BTS102 | 0.99 |
| 13BTS196 | 13BTS035 | 0.97 | 13BTS101 | 0.97 |
| 13BTS197 | - | - | - | - |
| 13BTS198 | 13BTS107 | 0.93 | 13BTS022 | 0.99 |
| 13BTS199 | 13BTS038 | - | 13BTS101 | 0.97 |
| 13BTS200 | 13BTS003 | - | 13BTS024 | - |
| 13BTS201 | - | - | 13BTS069 | 0.97 |
| 13BTS202 | - | - | 13BTS031 | 0.97 |
| 13BTS203 | 13BTS010 | 0.97 | 13BTS101 | 0.97 |
| 13BTS204 | 13BTS125 | 0.93 | 13BTS084 | - |
| 13BTS205 | 13BTS013 | 0.97 | 13BTS102 | 0.99 |
| 13BTS206 | 13BTS125 | 0.93 | 13BTS102 | 0.99 |
| 13BTS207 | 13BTS098 | 0.97 | 13BTS023 | 0.99 |
| 13BTS208 | 13BTS016 | 0.93 | 13BTS022 | 0.99 |
| 13BTS209 | 13BTS096 | 0.93 | 13BTS022 | 0.99 |
| 13BTS210 | - | - | 13BTS073 | 0.99 |
| 13BTS214 | 13BTS050 | - | 13BTS102 | 0.99 |
| 13BTS215 | 13BTS055 | - | 13BTS037 | 0.99 |
| 13BTS216 | 13BTS115 | 0.93 | 13BTS036 | 0.99 |
| 13BTS217 | 13BTS009 | 0.97 | 13BTS101 | 0.97 |
| 13BTS218 | 13BTS079 | 0.97 | 13BTS031 | 0.97 |
| 13BTS219 | - | - | - | - |
| 13BTS220 | 13BTS012 | - | 13BTS069 | 0.97 |
| 13BTS221 | 13BTS133 | 0.97 | 13BTS101 | 0.97 |
| 13BTS222 | - | - | 13BTS022 | 0.99 |
| 13BTS223 | - | - | 13BTS101 | 0.97 |
| 13BTS224 | 13BTS020 | 0.97 | 13BTS069 | 0.97 |
| 13BTS225 | 13BTS109 | 0.97 | 13BTS031 | 0.97 |
| 13BTS226 | - | - | 13BTS037 | 0.99 |
| 13BTS227 | 13BTS056 | 0.97 | 13BTS031 | 0.97 |
| 13BTS230 | 13BTS003 | - | 13BTS051 | - |
| 13BTS231 | - | - | 13BTS101 | 0.97 |
| 13BTS232 | 13BTS075 | 0.97 | - | - |
| 13BTS234 | 13BTS075 | 0.97 | 13BTS073 | 0.99 |
| 13BTS235 | - | - | 13BTS005 | 0.99 |
| 13BTS236 | 13BTS065 | 0.93 | - | - |
| 13BTS237 | 13BTS060 | 0.93 | 13BTS073 | 0.99 |
| 13BTS238 | 13BTS079 | 0.97 | 13BTS073 | 0.99 |
| 13BTS239 | 13BTS064 | - | 13BTS072 | - |
| 13BTS240 | - | - | 13BTS101 | 0.97 |
| 13BTS241 | 13BTS060 | 0.93 | 13BTS087 | 0.99 |
| 13BTS242 | 13BTS010 | 0.97 | 13BTS101 | 0.97 |
| 13BTS243 | - | - | 13BTS022 | 0.99 |
| 13BTS244 | 13BTS009 | 0.97 | 13BTS101 | 0.97 |
| 13BTS246 | 13BTS060 | - | 13BTS073 | - |
| 13BTS247 | 13BTS010 | 0.97 | 13BTS101 | 0.97 |
| 13BTS248 | 13BTS003 | 0.97 | 13BTS006 | 0.99 |
| 13BTS249 | 13BTS021 | 0.97 | 13BTS031 | 0.97 |
| 13BTS253 | 13BTS015 | 0.93 | 13BTS031 | 0.97 |
| 13BTS254 | 13BTS064 | 0.93 | 13BTS072 | 0.99 |
| 13BTS255 | 13BTS009 | 0.97 | 13BTS101 | 0.97 |
| 13BTS256 | - | - | 13BTS087 | 0.99 |
| 13BTS257 | 13BTS133 | 0.97 | 13BTS046 | 0.97 |
| 13BTS258 | 13BTS107 | - | 13BTS022 | - |
| 13BTS259 | - | - | 13BTS086 | 0.99 |
| 13BTS260 | - | - | 13BTS101 | - |
| 13BTS261 | 13BTS125 | 0.93 | - | - |
| 13BTS262 | 13BTS015 | 0.93 | 13BTS031 | 0.97 |
| 13BTS263 | - | - | - | - |
| 13BTS264 | - | - | 13BTS006 | 0.99 |
| 13BTS265 | 13BTS049 | 0.97 | 13BTS087 | 0.99 |
| 13BTS267 | - | - | 13BTS084 | - |
| 13BTS268 | 13BTS010 | 0.97 | 13BTS054 | - |
| 13BTS269 | 13BTS010 | 0.97 | 13BTS052 | - |
| 13BTS270 | - | - | 13BTS031 | 0.97 |
| 13BTS271 | - | - | - | - |
| 13BTS272 | - | - | 13BTS101 | - |
| 13BTS273 | 13BTS065 | 0.93 | 13BTS091 | 0.99 |
| 13BTS274 | 13BTS065 | 0.93 | 13BTS091 | 0.99 |
| 13BTS276 | - | - | - | - |
| 13BTS278 | 13BTS048 | - | 13BTS086 | 0.99 |
| 13BTS280 | 13BTS065 | 0.93 | 13BTS091 | 0.99 |
| 13BTS281 | 13BTS125 | - | 13BTS037 | - |
| 13BTS283 | 13BTS114 | 0.97 | 13BTS077 | 0.99 |
| 13BTS284 | - | - | 13BTS046 | 0.97 |
| 13BTS285 | 13BTS133 | 0.97 | 13BTS046 | 0.97 |
| 13BTS287 | 13BTS060 | 0.93 | 13BTS073 | 0.99 |
| 13BTS288 | 13BTS189 | 0.93 | 13BTS141 | 0.99 |
| 13BTS289 | - | - | - | - |
| 13BTS290 | - | - | - | - |
| 13BTS291 | 13BTS033 | 0.93 | 13BTS058 | 0.97 |
| 13BTS293 | - | - | - | - |
| 13BTS294 | - | - | - | - |
| 13BTS295 | 13BTS033 | 0.93 | 13BTS058 | 0.97 |
| 13BTS296 | - | - | 13BTS031 | 0.97 |
| 13BTS297 | - | - | 13BTS101 | 0.97 |
| 13BTS298 | - | - | 13BTS002 | 0.99 |
| 13BTS299 | 13BTS010 | 0.97 | 13BTS101 | 0.97 |
| 13BTS301 | 13BTS107 | 0.93 | 13BTS022 | 0.99 |
| 13BTS302 | 13BTS075 | 0.97 | 13BTS023 | 0.99 |
| 13BTS303 | - | - | 13BTS087 | 0.99 |
| 13BTS304 | - | - | 13BTS002 | 0.99 |
| 13BTS305 | - | - | - | - |
| 13BTS308 | 13BTS114 | 0.97 | 13BTS077 | 0.99 |
| 13BTS310 | - | - | 13BTS006 | 0.99 |
| 13BTS311 | 13BTS015 | 0.93 | 13BTS031 | 0.97 |
| 13BTS315 | - | - | 13BTS058 | 0.97 |
| 13BTS316 | 13BTS096 | 0.93 | 13BTS022 | 0.99 |
| 13BTS317 | 13BTS081 | - | 13BTS058 | - |
| 13BTS318 | - | - | 13BTS054 | - |
| 13BTS320 | - | - | - | - |
| 13BTS322 | - | - | 13BTS037 | 0.99 |
| 13BTS323 | 13BTS174 | 0.97 | 13BTS260 | 0.97 |
| 13BTS324 | 13BTS159 | 0.93 | 13BTS260 | 0.97 |
| 13BTS325 | 13BTS081 | 0.97 | 13BTS058 | 0.97 |
| 13BTS326 | 13BTS119 | 0.97 | 13BTS022 | 0.99 |
| 13BTS328 | - | - | 13BTS162 | 0.99 |
| 13BTS332 | 13BTS189 | 0.93 | 13BTS141 | 0.99 |
| 13BTS334 | 13BTS029 | 0.97 | 13BTS102 | 0.99 |
| 13BTS338 | - | - | 13BTS162 | 0.99 |
| 13BTS339 | - | - | - | - |
| 13BTS342 | 13BTS159 | 0.93 | 13BTS260 | 0.97 |
| 13BTS343 | 13BTS159 | 0.93 | 13BTS162 | 0.99 |
| 13BTS346 | 13BTS174 | 0.97 | 13BTS162 | 0.99 |
| 13BTS348 | 13BTS174 | 0.97 | 13BTS162 | 0.99 |
| 13BTS349 | - | - | - | - |
| 13BTS351 | - | - | 13BTS141 | 0.99 |
| 13BTS352 | 13BTS207 | 0.97 | 13BTS162 | 0.99 |
| 13BTS359 | 13BTS287 | 0.97 | 13BTS311 | 0.99 |
| 13BTS360 | - | - | 13BTS246 | 0.97 |
| 13BTS361 | 13BTS270 | 0.97 | 13BTS206 | 0.99 |
| 13BTS362 | 13BTS191 | 0.97 | 13BTS141 | 0.99 |
| 13BTS363 | 13BTS189 | 0.93 | 13BTS141 | 0.99 |
| 13BTS364 | 13BTS278 | 0.97 | 13BTS200 | 0.97 |
| 13BTS365 | 13BTS270 | 0.97 | 13BTS162 | 0.99 |
| 13BTS366 | 13BTS255 | 0.97 | 13BTS281 | 0.97 |
| 13BTS367 | 13BTS239 | 0.93 | 13BTS140 | 0.99 |
| 13BTS368 | 13BTS189 | 0.93 | 13BTS141 | 0.99 |
| 13BTS369 | 13BTS189 | 0.93 | 13BTS141 | 0.99 |
| 13BTS370 | - | - | 13BTS190 | 0.99 |
| 13BTS371 | - | - | 13BTS162 | 0.99 |
| 13BTS372 | 13BTS222 | 0.97 | 13BTS162 | 0.99 |
| 13BTS373 | 13BTS189 | 0.93 | 13BTS141 | 0.99 |
| 13BTS374 | 13BTS339 | 0.93 | 13BTS141 | 0.99 |
| 13BTS377 | 13BTS174 | 0.97 | 13BTS162 | 0.99 |
| 13BTS378 | - | - | - | - |
| 13BTS379 | 13BTS339 | 0.93 | 13BTS260 | 0.97 |
| 13BTS381 | 13BTS339 | 0.93 | 13BTS141 | 0.99 |
| 13BTS382 | 13BTS296 | 0.97 | 13BTS141 | 0.99 |
| 13BTS383 | 13BTS134 | 0.97 | 13BTS162 | 0.99 |
| 13BTS384 | 13BTS270 | 0.97 | 13BTS236 | 0.99 |
| 13BTS385 | 13BTS287 | 0.97 | 13BTS258 | 0.97 |
| 13BTS388 | 13BTS278 | 0.97 | 13BTS200 | 0.97 |
| 13BTS391 | 13BTS134 | 0.97 | 13BTS162 | 0.99 |
| 13BTS395 | 13BTS255 | 0.97 | 13BTS140 | 0.99 |
| 13BTS398 | 13BTS272 | 0.93 | 13BTS311 | 0.99 |
| 13BTS399 | 13BTS255 | 0.97 | 13BTS325 | 0.99 |
| 13BTS400 | 13BTS204 | 0.97 | 13BTS260 | 0.97 |
| 13BTS402 | 13BTS174 | 0.97 | 13BTS162 | 0.99 |
| 13BTS403 | 13BTS540 | 0.97 | 13BTS140 | 0.99 |
| 13BTS404 | 13BTS255 | 0.97 | 13BTS281 | 0.97 |
| 13BTS405 | - | - | 13BTS140 | 0.99 |
| 13BTS406 | 13BTS272 | 0.93 | 13BTS140 | 0.99 |
| 13BTS407 | 13BTS174 | 0.97 | 13BTS162 | 0.99 |
| 13BTS408 | 13BTS339 | 0.93 | 13BTS260 | 0.97 |
| 13BTS409 | - | - | 13BTS141 | 0.99 |
| 13BTS410 | 13BTS222 | 0.97 | 13BTS162 | 0.99 |
| 13BTS413 | 13BTS134 | 0.97 | 13BTS162 | 0.99 |
| 13BTS414 | 13BTS222 | 0.97 | 13BTS260 | 0.97 |
| 13BTS417 | - | - | 13BTS246 | 0.97 |
| 13BTS419 | 13BTS159 | 0.93 | 13BTS141 | 0.99 |
| 13BTS421 | 13BTS296 | 0.97 | 13BTS141 | 0.99 |
| 13BTS422 | 13BTS193 | 0.93 | 13BTS260 | 0.97 |
| 13BTS423 | 13BTS540 | 0.97 | 13BTS267 | - |
| 13BTS425 | 13BTS317 | - | 13BTS258 | 0.97 |
| 13BTS429 | - | - | 13BTS162 | 0.99 |
| 13BTS430 | - | - | 13BTS162 | 0.99 |
| 13BTS431 | 13BTS222 | 0.97 | 13BTS140 | 0.99 |
| 13BTS436 | 13BTS191 | 0.97 | 13BTS140 | 0.99 |
| 13BTS439 | - | - | 13BTS260 | - |
| 13BTS440 | - | - | 13BTS162 | 0.99 |
| 13BTS441 | 13BTS193 | 0.93 | 13BTS140 | 0.99 |
| 13BTS442 | 13BTS339 | - | 13BTS141 | - |
| 13BTS444 | - | - | - | - |
| 13BTS445 | 13BTS159 | 0.93 | 13BTS246 | 0.97 |
| 13BTS457 | 13BTS134 | 0.97 | 13BTS162 | 0.99 |
| 13BTS460 | - | - | 13BTS236 | 0.99 |
| 13BTS468 | 13BTS222 | 0.97 | 13BTS140 | 0.99 |
| 13BTS472 | 13BTS261 | 0.97 | 13BTS260 | 0.97 |
| 13BTS500 | - | - | - | - |
| 13BTS501 | 13BTS517 | 0.97 | 13BTS325 | 0.99 |
| 13BTS503 | - | - | - | - |
| 13BTS504 | 13BTS255 | 0.97 | 13BTS140 | 0.99 |
| 13BTS510 | 13BTS193 | 0.93 | 13BTS140 | 0.99 |
| 13BTS514 | 13BTS235 | 0.97 | 13BTS190 | 0.99 |
| 13BTS517 | - | - | 13BTS162 | 0.99 |
| 13BTS518 | - | - | - | - |
| 13BTS521 | 13BTS372 | 0.97 | 13BTS527 | - |
| 13BTS525 | - | - | - | - |
| 13BTS526 | - | - | 13BTS260 | 0.97 |
| 13BTS527 | 13BTS339 | - | 13BTS141 | - |
| 13BTS529 | 13BTS189 | 0.93 | 13BTS141 | 0.99 |
| 13BTS532 | - | - | 13BTS141 | 0.99 |
| 13BTS535 | 13BTS278 | 0.97 | 13BTS200 | 0.97 |
| 13BTS540 | 13BTS033 | 0.93 | 13BTS121 | 0.99 |
| 13BTS543 | - | - | - | - |
| 13BTS548 | - | - | - | - |
| 13BTS549 | - | - | - | - |
| 13BTS551 | 13BTS526 | 0.97 | 13BTS529 | 0.99 |
| 13BTS553 | 13BTS056 | 0.97 | 13BTS031 | 0.97 |
| 13BTS556 | - | - | 13BTS162 | 0.99 |
| 13BTS557 | 13BTS174 | 0.97 | 13BTS162 | 0.99 |
| 13BTS558 | 13BTS207 | 0.97 | 13BTS162 | 0.99 |
| 13BTS559 | - | - | - | - |
| 13BTS561 | 13BTS272 | 0.93 | 13BTS140 | 0.99 |
| 13BTS562 | 13BTS287 | 0.97 | 13BTS258 | 0.97 |
| 13BTS563 | 13BTS174 | 0.97 | 13BTS162 | 0.99 |
| 13BTS565 | 13BTS278 | 0.97 | 13BTS200 | 0.97 |
| 13BTS566 | 13BTS189 | 0.93 | 13BTS141 | 0.99 |
| 13BTS571 | - | - | 13BTS260 | 0.97 |
| 13BTS574 | 13BTS191 | 0.97 | 13BTS140 | 0.99 |
| 13BTS575 | 13BTS193 | 0.93 | 13BTS162 | 0.99 |
| 13BTS576 | 13BTS272 | - | 13BTS162 | - |
| 13BTS577 | 13BTS566 | 0.97 | 13BTS576 | - |
| 13BTS588 | - | - | - | - |
| 13BTS591 | 13BTS272 | 0.93 | 13BTS162 | 0.99 |
| 13BTS597 | 13BTS239 | 0.93 | 13BTS260 | 0.97 |
| 13BTS598 | 13BTS239 | 0.93 | 13BTS140 | 0.99 |
| 13BTS601 | 13BTS372 | 0.97 | 13BTS608 | 0.99 |
| 13BTS602 | - | - | - | - |
| 13BTS603 | 13BTS429 | 0.97 | 13BTS377 | 0.99 |
| 13BTS605 | 13BTS439 | 0.93 | 13BTS377 | 0.99 |
| 13BTS607 | 13BTS566 | 0.97 | 13BTS518 | - |
| 13BTS608 | - | - | 13BTS162 | 0.99 |
| 13BTS609 | 13BTS328 | 0.97 | 13BTS442 | 0.97 |
| 13BTS620 | 13BTS270 | 0.97 | 13BTS236 | 0.99 |
| 13BTS621 | - | - | - | - |
| 13BTS625 | - | - | - | - |
| 13BTS630 | - | - | 13BTS608 | 0.99 |
| 13BTS634 | 13BTS372 | 0.97 | 13BTS608 | 0.99 |
| 13BTS640 | 13BTS272 | 0.93 | 13BTS236 | 0.99 |
| 13BTS641 | 13BTS368 | 0.97 | 13BTS442 | 0.97 |
| 13BTS643 | 13BTS439 | 0.93 | 13BTS377 | 0.99 |
| 13BTS649 | 13BTS261 | 0.97 | - | - |
| 13BTS662 | 13BTS526 | 0.97 | 13BTS529 | 0.99 |
| 13BTS664 | 13BTS526 | 0.97 | 13BTS529 | 0.99 |
| 13BTS670 | - | - | 13BTS267 | - |
